# 

**Published:** 1889-09-07

**Authors:** 


SATURDAY, SEPTEMBER 7th, 1889.

**Originality of Method** - 351
**Words of Consolation**—Awakening - 352
**The Doctor's Pulpit.**—In Nature's School - 353
**The Pleasures of Imagination Defended** - 354
**The Story of the Insane from Year to Year.**—Asylum Reports - 354
**Within the Wards.**—Beef-Tea and how to make it - 355
**The Boarding Out of Pauper Children.** I.—The System Itself - 356
**Children's Country Holidays Fund** - 356
**On Some Points Connected with Cholera** - 357
**Notes and News** - 358
**Everybody's Page** - 360
**Annotations.**—What is the Next Step?—"The Law of Battle"—Disease and Will-Making - 361
**The Plans and Purposes of the Johns Hopkins Hospital.** IV.—The Hospital and Medical Education. By JOHN S. BILLINGS, M.D., Surgeon, U.S. Army - 362
**Her Heart's Mistake.** By E. D. CUMMING. Chap. xxi. - 363
**The Hospital Workshop.** IX.—Out-Patients at St. Bartholomew's - 365
**Amusements—Scraps and Gleanings** - 366

**EXTRA SUPPLEMENT—THE NURSING MIRROR.**

**En Passant.**—Luton Cottage Hospital—A Women's Parade—Birmingham Hospital—Miss Whately's Work—New York Nursing—Nurses at Rome—An Eccentric Sister—Male Nurses—Examination Questions - lxxxvii
**Physiology for Nurses.** No. 8.—Joints and Muscles lxxxviii
**Nurses on their Travels.**—Across Canada - lxxxix
**The General Hospital, Birmingham.**—Speech of the Chairman before Distributing Medals to Nurses - lxxxix
**Everybody's Opinion.**—The Pension Fund—Male Nurses - xc
**Presentation** - xc
**Notes and Queries** - xc
**Appointments—Vacancies** - xc

